# Effect of oleaster (*Elaeagnus angustifolia* L.) flour addition combined with high‐pressure homogenization on the acidification kinetics, physicochemical, functional, and rheological properties of kefir

**DOI:** 10.1002/fsn3.3491

**Published:** 2023-06-09

**Authors:** Latife Betül Gül, Saya Bekbay, Abdullah Akgün, Osman Gül

**Affiliations:** ^1^ Department of Food Engineering Faculty of Engineering Giresun University Giresun Turkey; ^2^ Department of Food Technology and Processing Products Technical Faculty Saken Seifullin Kazakh AgroTechnical Research University Nur‐Sultan (Astana) Kazakhstan; ^3^ Department of Food Engineering Faculty of Engineering Trakya University Edirne Turkey; ^4^ Department of Food Engineering Faculty of Engineering and Architecture Kastamonu University Kastamonu Turkey

**Keywords:** functional foods, high‐pressure homogenization, kefir, oleaster flour, rheology, structure

## Abstract

In this study, the effects of peeled oleaster flour (OF) addition (0.5% and 1%) with high‐pressure homogenization (HPH) at 100 MPa on acidification kinetics, physicochemical, functional, and rheological properties of kefir made from bovine whole milk were investigated. The fermentation kinetic parameters such as *Vmax* and *T*_*f*_ decreased by 23.63% and 20%, respectively, with 1% OF and application of HPH. The combined use of two treatments had a positive effect on *Lactobacillus* and *Lactococcus* counts, reaching a maximum of 9.63 and 9.31 log cfu/mL, respectively. Also, total phenolic contents and antioxidant activity reached maximum values of 85.31 mg GAE/g and 17.22%, respectively. The *ΔE* value was more limited with HPH. The maximum firmness and water‐holding capacity values were determined in the sample produced with 1% OF and application of HPH. Rheological analysis revealed that all kefirs exhibited shear thinning behavior, and the Ostwald–de‐Waele (*R* ^2^ > .99) model was suitable to describe the rheological behavior of all kefir samples. The highest viscosity (0.049 Pa.s, at 50/s shear rate) and consistency index (1.115 Pa.s^*n*^) were observed in kefir with 1% OF and application of HPH. Kefir samples were characterized as weak gel behavior because storage modulus (G') was much greater than loss modulus (G") and the power‐law model was used to characterize the viscoelasticity. The overall quality assessment indicated that the improvement of the fermentation process and the enhancement of textural and functional properties of kefir samples could be achieved with the addition of 1% OF and application of HPH.

## Graphical abstract

The use of OF combined with the HPH treatment to improve functional, structural, and rheological properties as well as fermentation kinetics of kefir was investigated. The combined use of two treatments can decrease the fermentation time by approximately 4 h compared to the control sample. Bioactive properties were improved by the use of OF, and HPH contributed to this increase. Both methods also play an important role in improving the physical and rheological properties and water‐holding capacity of kefir.

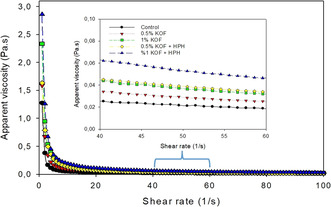

## 1 INTRODUCTION

Kefir, which originates from the Caucasus mountains, Tibet, and Mongolia, is a slightly carbonated fermented milk beverage with an acidic taste (pH 4.6) and a creamy consistency, containing a small amount of alcohol (Aiello et al., 2020; Irigoyen, 2005). From Japan to Eastern and Northern Europe, it has become a widely consumed beverage, due to its nutritional value and health benefits such as reducing lactose intolerance symptoms, stimulating the immune system, and cholesterol‐lowering, antimutagenic, and anti‐carcinogenic properties (Gul et al., 2015; Paredes et al., 2022). Kefir is produced using kefir grain, kefir starter culture, or commercial lyophilized starter culture containing a mixture of lactic and acetic acid bacteria and yeasts (Gul et al., 2018). It has a unique flavor and taste due to the diversity of microorganisms and the symbiotic relationship it contains (Sözeri Atik et al., 2021). Lactic acid, CO_2_, acetaldehyde, acetoin, diacetyl, and ethanol are the main fermentation end products responsible for the aroma of kefir (Beshkova et al., 2003; Güzel‐Seydim et al., 2000). In addition, various vitamins (such as vitamins B_1_, B_9_, B_12_, and K), amino acids, and calcium increase in kefir during fermentation (Otles & Cagindi, 2003) and also different bioactive compounds such as peptides with beneficial effects on health and heteropolysaccharides such as kefiran are formed (Kim et al., 2019).

Recently, the increasing demand of consumers for healthy foods is due to their medical reasons or lifestyle choices. This promotes the studies on the usage of plant‐based ingredients or materials with health benefits in kefir. Moreover, this approach, which is taken into account to increase the nutraceutical benefits of kefir, also plays a role in the improvement/change in the structural properties of the product. For this purpose, the addition of inulin (Glibowski & Kowalska, 2012), water‐soluble extracts (Alves et al., 2021), and honey (Doğan, 2010), and also the use of plant‐based beverages in kefir production (Atalar, 2019; Santos et al., 2019) are included in the literature. Moreover, there have been many studies published recently about potential of oleaster to enhance the functional and structural properties of various dairy products and beverages (Çakmakçı et al., 2015; Darvishzadeh et al., 2021; Öztürk et al., 2018). Oleaster (*Elaeagnus angustifolia* L.), known as the Russian olive, grows in very coarse climatic conditions in many countries including Turkey (Yavuz et al., 2021). Oleaster fruits and seeds are very important for health due to great sources of natural antioxidants and can be used as diuretic, tonic, antipyretic, and antidiarrhea, in the prevention of intestinal disorders, and as an anti‐inflammatory and analgesic in traditional medicine (Çakmakçı et al., 2015; Sahan et al., 2015). Additionally, it is used in the development of functional products due to its floury structure, specific taste, and functional properties such as phenolic compounds, dietary fiber, and mineral content (Yasemin Sahan et al., 2013). Moreover, it is recommended to be used as a thickening or functional agent in various food systems such as yoghurt, ice cream, beverages, and bakery products because of its functional properties (such as solubility and water‐holding capacity; Sahan et al., 2015). According to Çakmakçı et al. (2015), it was reported that the addition of oleaster flour and crust positively affected the sensory properties, increased the sweetness of the ice cream, and the ice cream containing 2% OF received the highest score from the panelists. In another study, it was determined that the fermentation time of yoghurt samples enriched with 2% OF was significantly shortened, their water‐holding capacity was improved, and thus their syneresis decreased during storage (Öztürk et al., 2018).

Another approach to improving the structural and functional properties of foods is the use of novel continuous technology such as high‐pressure homogenization (HPH). HPH is a promising technology that operates at significantly higher pressures (up to 350 MPa) and is being researched to improve the properties of food and food ingredients, especially dairy products (Atalar, 2019; Levy et al., 2021; Serra et al., 2009; Tsevdou et al., 2020). Rodarte et al. (2018) reported that the total bacterial loads of ice cream samples treated with high pressure were lower than the samples treated with convectional homogenization, and physicochemical properties such as particle size and stability were improved by HPH technology. In another study, it was determined that the G′ and G′′ values of fermented camel milk were enhanced by HPH treatment and HPH was a more effective method in the development of rheological properties of fermented beverage compared to the heat treatment and starter culture (Ayyash et al., 2022).

For the improvement of functional properties of dairy products, it is known that some structural and sensory problems occur with the use of plant‐based additives or materials with high fiber content in dairy products. Öztürk et al. (2018) notified that the more soft gel occurs in yoghurt sample with the usage of OF contain fibers due to their dispersion in casein micelles. On the other hand, sensory properties such as overall flavor and texture are negatively affected by the usage of fibers, including intense grainy flavor and gritty texture (Fernandez‐Garcia & McGregor, 1997; Tomic et al., 2017). Combination with HPH as a new technology has an important potential in eliminating these problems caused by the use of plant‐based materials with high fiber content to improve the functional properties of dairy products. As far as we know, there is no study on the incorporation of OF with a combination of HPH treatment. The study aimed to evaluate the use of OF and HPH treatment to improve the physicochemical, rheological, and functional properties of kefir. In addition, the effect of OF and HPH on the fermentation kinetics of kefir was also determined.

## 2 MATERIALS AND METHODS

### 2.1 Material

Commercial milk (Migros brand; 11.68% total solid, 3.18% protein, 3.25% fat, and 0.64% ash, 6.62 pH) was used in the production of kefir samples. Commercial freeze‐dried kefir starter culture (Sevdanem brand) was supplied by Danem Ltd., which contains *Lactobacillus kefiri*, *L. parakefiri*, *L. acidophilus*, *L. kefiranofaciens* subsp. *kefirgranum*, *L. bulgaricus*, *L. casei*, *L. fermentum*, *L. reuteri*, *L. helveticus*, *L. plantarum*, *Leuconostoc mesentereoides*, *Streptococcus thermophiles*, *Lactococcus lactis* subsp. *lactis*, *Bifidobacterium bifidum*, *Kluyveromyces marxianus*, and *Saccharomyces cerevisiae*. Dried oleaster (*Elaeagnus angustifolia* L.) fruits were provided by Komşu Agricultural Products Inc. To obtain peeled OF, the fruit flesh was dried using a freeze drier (Teknosem, Toros TRS‐4/4) after peeling and deseeding, and then ground using an electric powder grinder to get micro‐size powders. The chemicals used in this study were obtained from Sigma Chemical Co. and Merck.

### 2.2 Kefir production

For kefir manufacture, milk was divided into five experimental groups, which were control (without OF and HPH), 0.5% and 1% OF added, and 0.5% and 1% OF added and HPH treated. HPH treatment at 100 MPa was carried out after the OF addition using a high‐pressure homogenizer (Panda Plus 2000, GEA Niro Soavi). The temperature of milk before homogenization was 8°C, and it was determined as 12°C after the homogenization process. After the addition of OF and homogenization, the milk was pasteurized at 90°C for 10 min using a water bath and rapidly cooled down to 25°C. Inoculation was done with freeze‐dried kefir starter culture at a level of 0.5 g/L, and then the samples were incubated at 25°C until the pH reached ~4.5. The kefir samples produced were stored at 4°C until further analysis.

### 2.3 Proximate analysis

The pH values of kefir were measured using a pH meter (Hanna edge, Hanna Instruments) after calibration with standard buffers with pH 4.0, 7.0, and 10.0. Titratable acidity in terms of % lactic acid was determined by titrating with 0.1 N NaOH. The total solids, fat, protein, and ash contents of samples were determined by the AOAC official method (AOAC, 1992).

### 2.4 Microbiological analysis

An aliquot (10 mL) of the sample was dispersed with 90 mL of sterile saline buffer (0.85% NaCl), homogenized with a stomacher for 60 s, and decimal dilutions were prepared. Lactobacilli populations were counted using MRS Agar (Merck) after incubation under anaerobic conditions at 30°C for 48 h. M17 agar (Merck) was used to determine the lactococci after incubation at 30°C for 72 h (Gul et al., 2015). To measure bacterial counts, the pour plate technique was used. The results were presented in log cfu/mL.

### 2.5 Fermentation kinetics

pH was measured every 2 h using a digital pH meter, and microbiology analyses were carried out every 4 h throughout the fermentation process. The acidification rate (*V*_max_, expressed as pH units/min) was determined as the time variation of pH (dpH/dt). The time to reach the maximum acidification rate (*T*_max_), time to reach pH 5.0 (*T*_5_), and time to complete the fermentation (*T*_*f*_) were determined as responses that characterized the kinetics of the fermentation process.

### 2.6 Color properties

Color measurement was performed by using a colorimeter (ColorFlex EZ colorimeter; Hunter Associates Laboratory, Inc.). The CIELab chromaticity coordinates were used to evaluate L* (lightness), a* (red–green), and b* (yellow–blue) by taking an average of five readings from each sample and also total color difference (*ΔE*) was determined as follows (Equation 1):

(1) $$∆E={\left[{\left(∆L\right)}^{2}+{\left(∆a\right)}^{2}+{\left(∆b\right)}^{2}\right]}^{0.5}$$

### 2.7 Total phenolic contents and antioxidant activity

Before the determination of bioactive properties, the extraction of bioactive compounds from kefir samples was conducted following the method described by Sözeri Atik et al. (2021). Kefir samples (10 g) were transferred into a test tube that included 10 mL of 75% methanol solution and homogenized by an ultra‐turrax homogenizer. Thereafter, the mixtures were centrifuged (Nuve, NF 800R) at 8000*× g* for 30 min at 4°C, and supernatants were used for the determination of bioactive properties.

Total phenolic contents were determined according to Folin–Ciocalteau method. According to the method, 1 mL of extract was mixed with 2.5 mL of 0.2 N Folin–Ciocalteau phenol reagent and 2 mL of sodium carbonate. The absorbance of the mixture was read at 760 nm using a UV/VIS spectrophotometer (Shimadzu UV‐1800) after incubation for 30 min in the dark at room temperature. Total phenolic content was calculated as μg gallic acid equivalents (GAEs) per gram of sample based on plots of a gallic acid calibration curve (*R* ^2^ = .9998; Singleton & Rossi, 1965).

Antioxidant activity was determined based on the 2,2‐diphenyl‐1‐picrylhydrazyl radical scavenging activity (DPPH). For this, 0.1 mL of extract was diluted with 4.9 mL DPPH solution, and the mixture was incubated in the dark for 30 min at room temperature. The absorbance was recorded at 517 nm by a spectrometer, and results were expressed as % activity that was calculated using Equation 2 (Abdel‐Hamid et al., 2020). The “blank” formulation used water as the ABTS working solution instead of the sample.

(2) $$\%\text{Activity}=\left[100-\left(\frac{\text{Absorbance of sample}}{\text{Absorbance of Blank}}\right)\right]*100$$

### 2.8 Water‐holding capacity and firmness

Water‐holding capacity (WHC) of kefir samples was evaluated according to the method reported by Gul et al. (2018). Approximately 10 g of homogenized kefir sample was weighed into a test tube and subjected to 10 min centrifugation at 3250*× g* at 4°C. After centrifuge, the supernatant was weighed, and the percentage of WHC was calculated using Equation (3).

(3) $$\mathrm{WHC}\left(\%\right)=\left(1-\frac{W_{1}}{W_{2}}\right)x100$$

where *W*_*1*_ and *W*_*2*_ are the weight of whey after centrifugation and kefir, respectively.

The textural measurements of kefirs were performed using a TA.HD Plus Texture Analyzer (Stable Micro Systems) equipped with a cylindrical probe (1/2″ diameter) and an extension bar having a 5 kg load cell. The test was replicated three times at a pretest speed of 1 mm/s and a test speed of 1 mm/s through 15 mm within the samples. After the temperature was allowed to equilibrate at 20°C, test was done and the firmness was defined as a maximal peak value recorded after the first immersion into the sample.

### 2.9 Rheological analysis

#### 2.9.1 Steady shear tests

The rheological measurements of kefirs were performed using a Haake Mars III rheometer (Thermo Scientific) equipped with a temperature controller unit. The cone‐plate geometry consisting of a 2° cone angle‐plate diameter of 35 mm with a gap size of 0.105 mm was used. After transferring the homogenized kefir sample to the bottom plate of the rheometer, the lift moved and the top plate took the measuring position. All measurements were carried out at a constant temperature of 25°C by recording shear stress against the shear rate range 0.0001–100 1/s. The dependence of viscosity on shear rate for all kefir samples was fitted to Ostwald–de Waele model (Equation 4).

(4) $${Ƞ}_{\mathrm{app}}=\mathrm{Kx}\gamma^{n-1}$$

where *η*_app_, *K, γ*, and *n* refer to the apparent viscosity (Pa.s), the consistency index (Pa.s^*n*^), the shear rate (1/s), and the flow behavior index (dimensionless), respectively.

#### 2.9.2 Dynamic shear test

To determine the viscoelastic properties of kefirs, linear viscoelastic region (LVR) was previously determined at a stress range between 0.01 and 10 Pa at a frequency of 1 Hz. After that, frequency sweep tests at 1 Pa (within the LVR) were performed at a frequency range of 0.1 and 100 Hz at 25°C. The storage (G′) and loss (G″) modules obtained were modeled as a power function of oscillatory frequency using Equations 5 and 6, respectively, which is commonly used to describe the viscoelastic behavior of food and dispersions.

(5) $$G^{\prime }=K^{\prime }{\left(⍵\right)}^{n^{\prime }}$$

(6) $$G^{\prime \prime }=K^{\prime \prime }{\left(⍵\right)}^{n^{\prime \prime }}$$

where, *K′* and *K″* are power‐law modulus constants (Pa.s^*n*^), *n’* and *n”* may be referred to as the frequency exponents, *⍵* is the angular frequency (rad/s).

### 2.10 Statistical analysis

All data presented in this study were analyzed with SPSS statistical software (SPSS Inc.). Statistical difference between the means was determined using one‐way ANOVA followed by Duncan's post hoc test at a significance level of 0.05. Each measurement was performed on triplicate and the results were reported as mean ± standard deviation.

## 3 RESULTS AND DISCUSSION

### 3.1 Acidification and microbiological growth during fermentation

**FIGURE 1:**
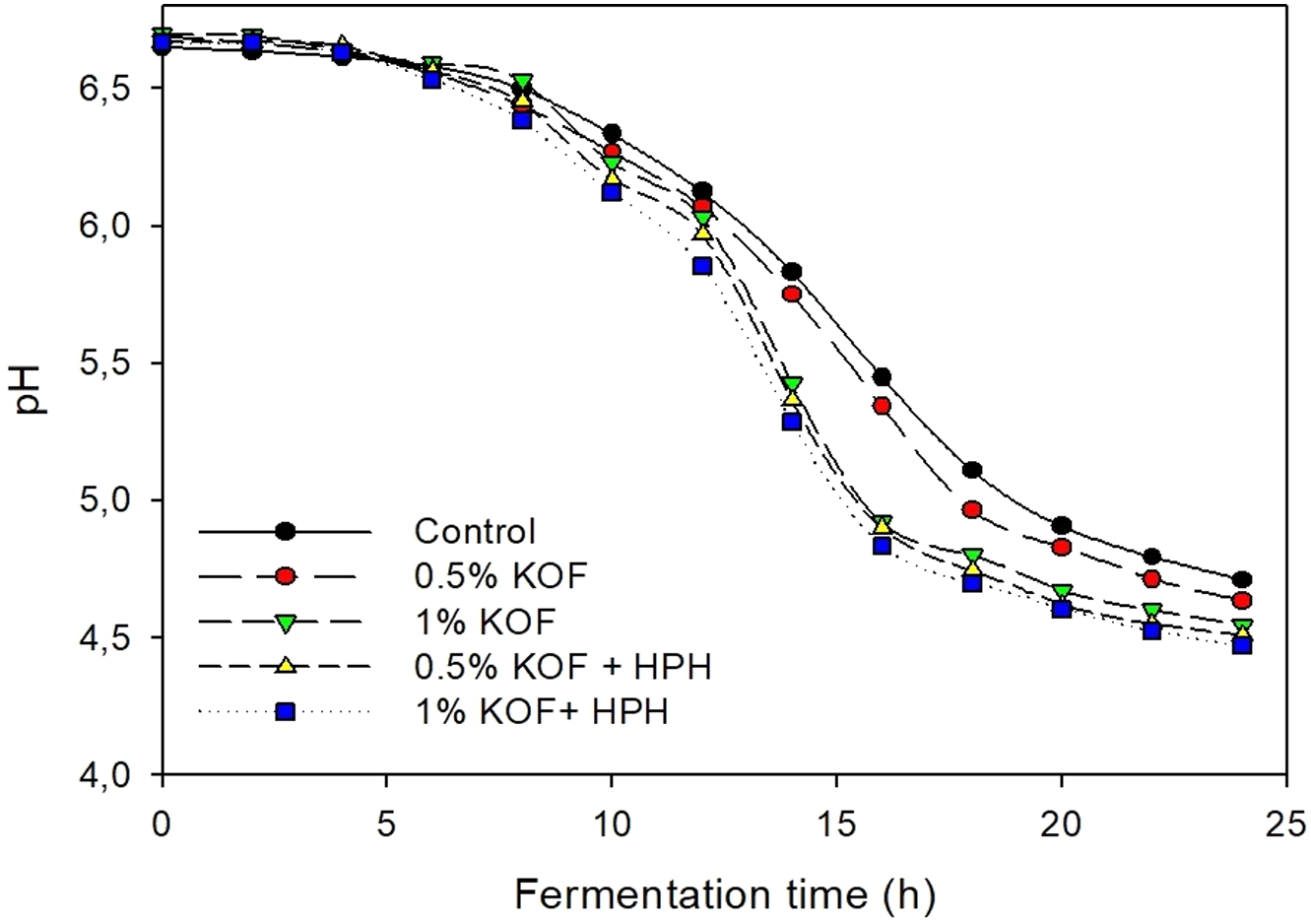
pH change during fermentation (0.5% KOF, kefir with 0.5% OF; 1% KOF, kefir with 1% OF; 0.5% KOF + HPH, kefir with 0.5% OF and application of HPH; 1% KOF + HPH, kefir with 1% OF and application of HPH).

The change in pH of kefir samples during fermentation period is shown in Figure 1, and the fermentation kinetic results (*V*_max_, *T*_max_ *, T*_5_, and *T*_*f*_) are given in Table 1. The pH change during fermentation was significantly affected depending on the added OF and the applied HPH, but there was no significant difference between the control and kefir with 0.5% OF. While the maximum acidification value was determined as 0.275 pH units/h in the control sample, it decreased to 0.21 pH units/h depending on the added OF and the applied HPH. The decrease in the maximum acidification value with OF is probably due to the buffering capacity of proteins, phenolic compounds, and organic acids such as citric and malic acids in oleaster. The buffering capacity of these compounds can lead to a decrease in *V*_max_ (do Espírito Santo et al., 2012). The lowest *Vmax* value was found in the sample with 1% OF and treated with HPH. The particle size decreases with the applied pressure, and therefore more components are released, resulting in higher buffering capacity. On the other hand, the time required to reach the *V*_max_ value was 12 h for the control and kefir with 0.5% OF. The addition of 1% OF and HPH treatment led to a decrease in *T*_max_ value for about 2 h. Similarly, *T*_5_ and *T*_*f*_ (the time required for the pH to decrease to 5 and 4.6, respectively) were longer in the control sample, and this time was reduced with OF and HPH application. Since the buffering capacity is directly inversely proportional to the total dry matter content of the fermented product, a prolonged fermentation period is necessary (Varghese & Mishra, 2008). However, this situation is incompatible with the increase in dry matter content with OF, and it is because the addition of OF increases the fermentation rate. Similar results were obtained for the enrichment of yogurt with passion fruit peel powder (do Espírito Santo et al., 2012) and OF (Öztürk et al., 2018). Öztürk et al. (2018) associated the reduction in fermentation time with OF containing dietary fiber (20.67%–30.65%), fructose (27.1%), and glucose (22.3%), which can be considered as an additional carbon source for microorganisms. Abdel‐Hamid et al. (2020) also stated that addition of *Siraitia grosvenorii* fruit extract to fermented product accelerated the fermentation process, which may include nutrients like proteins, carbohydrates, vitamins, and minerals that promote the growth of microorganisms responsible for the high acid production. On the other hand, fermentation rate is higher in the samples, especially those produced with HPH‐applied milk. The change in the distribution of water‐soluble and insoluble components such as calcium, phosphorus, and nitrogen in milk (even increasing with OF) during HPH application facilitates the usage of these components by starter cultures that play a role in fermentation, which can promote the activity and metabolism of cultures (Patrignani et al., 2016).

**TABLE 1:** Fermentation kinetic parameters of kefir samples.

Samples	*V*_max_ (pH units/h)	*T*_*max*_ (h)	*T*_5_ (h)	*T*_*f*_ (h)
Control	0.275 ± 0.001^a^	12	14	20
0.5% KOF	0.268 ± 0.008^a^	10	14	20
1% KOF	0.25 ± 0.001^b^	10	12	18
0.5% KOF + HPH	0.243 ± 0.008^c^	10	14	18
1% KOF + HPH	0.21 ± 0.005^d^	10	12	16

*Note*: Means in the same column with different lowercase letters show significant differences between samples (*p* < .05). *V*_max_, maximum rate of acidification; *T*_max_, time to reach *V*_max_; *T*_5_, time to reach pH 5.0; *T*_*f*_, time to reach pH 4.6. 0.5% KOF, kefir with 0.5% OF; 1% KOF, kefir with 1% OF; 0.5% KOF + HPH, kefir with 0.5% OF and application of HPH; 1% KOF + HPH, kefir with 1% OF and application of HPH.

**FIGURE 2:**
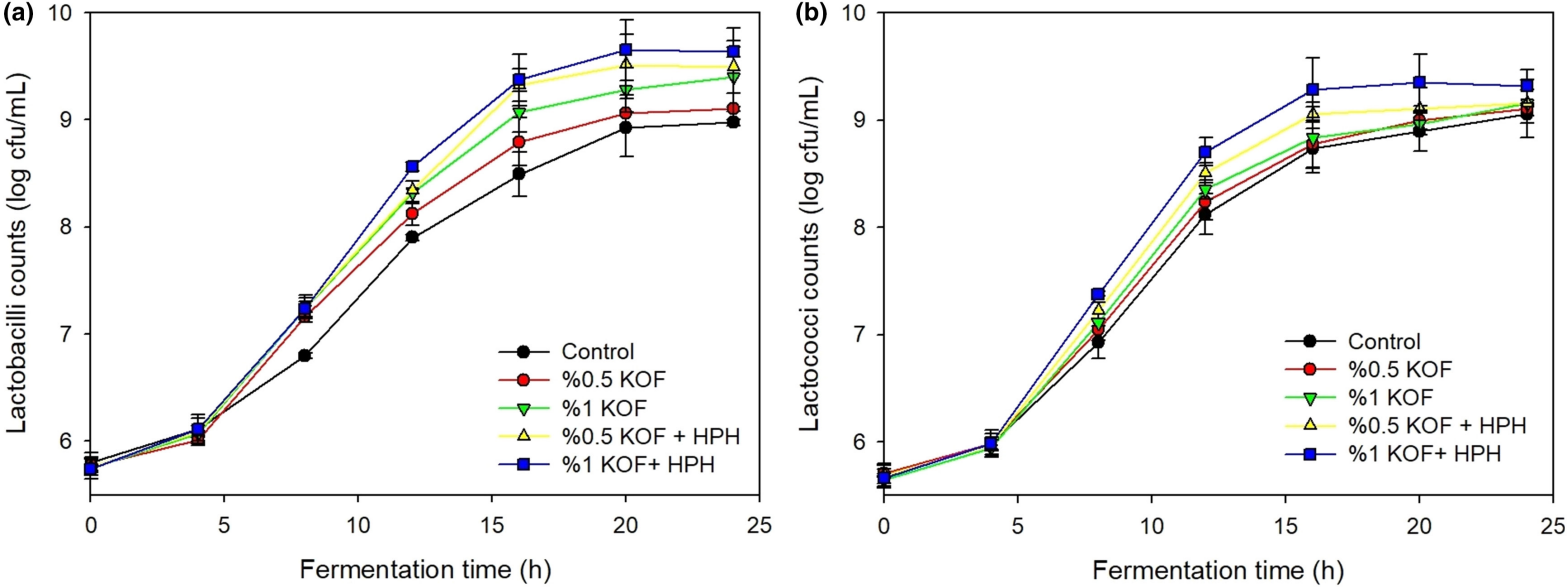
Lactobacilli and lactococci counts during fermentation (0.5% KOF, kefir with 0.5% OF; 1% KOF, kefir with 1% OF; 0.5% KOF + HPH, kefir with 0.5% OF and application of HPH; 1% KOF + HPH, kefir with 1% OF and application of HPH).

The bacterial growth in kefir samples during fermentation is given in Figure 2. At the end of the 24 h fermentation, lactobacilli and lactococci counts for control sample were found as 8.87 and 9.05 log cfu/mL, respectively. The addition of OF and the use of HPH in kefir production caused a rapid increase in cell viability in fermentation, and high viability level was reached at the end of fermentation. The highest number of lactobacilli (9.63 log cfu/mL) and lactococci (9.32 log cfu/mL) were determined in kefir sample with 1% OF and applied HPH. The addition of OF allowed the microorganisms to grow faster during fermentation. Although phenolic compounds have an antimicrobial effect, the addition of OF contributed significantly to the increase in cell viability in kefir samples. Possibly, the added OF concentration may not have been sufficient for the inhibitory effect on lactic acid bacteria. The increase in the number of microorganisms due to the addition of OF in kefir could be attributed to the increased availability of nutrients, which is supported by Darvishzadeh et al. (2021). Similarly, Ge et al. (2022) reported that sea buckthorn, which is used in the fortification of fermented milk beverages, promotes the growth of microorganisms. The homogenization process also contributed significantly to microbial growth. The water solubility of peeled OF is low due to its high starch content (Sahan et al., 2015), and the homogenization treatment probably caused a decrease in particle size and therefore, an increase in water solubility of OF. Consequently, accessibility to essential nutrients like carbon and nitrogen for bacteria and yeast growth can promote high levels of microbial growth (Darvishzadeh et al., 2021).

### 3.2 Proximate composition

Total solids, protein, ash, pH, and titratable acidity results of kefir samples are shown in Table 2. The total solid content of the control sample was found as 11.51%. As expected, the addition of OF led to an increment in total solids (*p* < .05), determined between 11.94% and 12.37%, depending on OF concentration. The findings of the current study are consistent with those of Öztürk et al. (2018) and do Espírito Santo et al. (2012), who reported that total solid contents increased with increasing additive concentration in the formulation. However, a partial increase was observed in protein and ash values, but it was not found to be statistically significant (*p* > .05), which is due to the relatively low protein (3.74%–4.65%) and ash (1.87%–2.46%) content of OF (Sahan et al., 2013). Taking into account the results of fermentation kinetics, pH values of kefir samples were measured between 4.48 and 4.68 after 20 h of fermentation. The addition of 1% OF with HPH treatment caused a rapid decrease in pH and fell below 4.5 at the end of the incubation period. Consistent with the pH value, the highest titratable acidity (0.93%) was determined in the kefir sample with 1% OF and HPH treatment. However, the lowest titratable acidity was determined in the control sample. Although the addition of OF caused an increase in the titratable acidity, the largest differences between the kefir samples with OF (except merely 1% KOF + HPH) were not detected (*p* > 0.05). Similar observations on the effects of peeled OF and passion fruit powder on pH and titratable acidity values of yogurt samples were stated by Öztürk et al. (2018) and do Espírito Santo et al. (2012), respectively.

**TABLE 2:** Physicochemical properties, total phenolics contents, and antioxidant activity of kefir samples.

	Control	0.5% KOF	1% KOF	0.5% KOF + HPH	1% KOF + HPH
Total solids (%)	11.51 ± 0.08^c^	11.96 ± 0.07^b^	12.36 ± 0.01^a^	11.94 ± 0.08^b^	12.36 ± 0.02^a^
Protein (%)	3.13 ± 0.07^a^	3.18 ± 0.1^a^	3.21 ± 0.07^a^	3.17 ± 0.06^a^	3.22 ± 0.11^a^
Ash (%)	0.61 ± 0.02^b^	0.68 ± 0.02^a^	0.72 ± 0.01^a^	0.67 ± 0.04^a^	0.7 ± 0.03^a^
pH	4.68 ± 0.01^a^	4.65 ± 0.01^b^	4.62 ± 0.01^c^	4.59 ± 0.02^d^	4.48 ± 0.02^e^
Titratable acidity (%)	0.79 ± 0.02^c^	0.87 ± 0.01^b^	0.88 ± 0.02^b^	0.89 ± 0.01^b^	0.93 ± 0.01^a^
L*	83.01 ± 0.11^a^	80.11 ± 0.38^c^	78.88 ± 0.85^d^	80.95 ± 0.52^bc^	81.08 ± 0.84^b^
a*	−1.29 ± 0.01^c^	−0.06 ± 0.05^b^	0.58 ± 0.06^a^	0.01 ± 0.03^b^	0.49 ± 0.09^a^
b*	6.28 ± 0.06^d^	6.64 ± 0.09^c^	7.44 ± 0.05^a^	6.37 ± 0.22^d^	7.11 ± 0.07^b^
*ΔE*	‐	3.17 ± 0.32^b^	4.69 ± 0.75^a^	2.36 ± 0.68^b^	2.85 ± 0.42^b^
Total phenolic contents (μg GAE/g)	60.83 ± 1.99^c^	64.85 ± 1.31^bc^	74.41 ± 2.76^ab^	76.53 ± 2.28^a^	83.31 ± 2.99^a^
DPPH (% activity)	5.33 ± 0.96^d^	5.65 ± 0.25^d^	6.83 ± 0.73^c^	12.41 ± 1.06^b^	17.22 ± 1.43^a^

*Note*: Means in the same row with different lowercase letters show significant differences between samples (*p* < .05). *ΔE*, total color difference, 0.5% KOF, kefir with 0.5% OF; 1% KOF, kefir with 1% OF; 0.5% KOF + HPH, kefir with 0.5% OF and application of HPH; 1% KOF + HPH, kefir with 1% OF and application of HPH.

### 3.3 Color properties

The color values (L*, a*, b*, and *ΔE*) of kefir samples are given in Table 2. The L*, a*, and b* values of the control sample were measured as 83.01, −1.29, and 6.28, respectively. The addition of OF caused a significant decrease in L* value and an increase in a* and b* values of kefirs (*p* < .05) when compared to the control. Similar results were also obtained by Çakmakçı et al. (2015) for ice cream and Öztürk et al. (2018) for yogurt, who indicated that brightness and greenness values are decreased while yellowness values are increased by the addition of OF that has between 78.91 and 82.59, 1.48 and 2.98, and 17.92 and 20.86 for L*, a*, and b*, respectively (Simsek & Sufer, 2021). Considering the unhomogenized kefir samples, a partial increase in L* values and a decrease in b* values of kefir samples were observed with the homogenization process. However, no change was detected in a* values. The color characteristics of the samples are related to the presence of insoluble particles that act as light‐scattering particles and color of the raw material used (Alves et al., 2021). After the homogenization process, the particles such as casein micelles, fat globules, and also OF particles get smaller, resulting in increase in the number of particles that can scatter light more effectively (Amador‐Espejo et al., 2014). Considering the *ΔE*, it was measured as 3.17 and 4.69 for kefir with 0.5% and 1% OF, respectively, and it was observed that the *ΔE* significantly decreased with the HPH application (*p* < .05). Therefore, the color characteristics of the kefir samples by the addition of OF were found to be close to the control sample by applying the homogenization process. Depending on the *ΔE* value, the differences in color compared to the control sample were determined to be lower than 3, indicating no detection by the human eye (Bernat et al., 2015).

### 3.4 Bioactive properties

Total phenolic contents of kefir samples were found between 60.82 and 83.31 μg GAE/g, as shown in Table 2. The lowest phenolic content was determined in the control sample and a significant increase was observed in the phenolic contents of kefir samples with OF (*p* < .05). Our results are compatible with studies on fortifying ice cream (Çakmakçı et al., 2015) and yogurt (Öztürk et al., 2018) with OF. Karkar and Şahin (2021) reported that the total phenolic content of OF after solvent extraction was between 0.13 and 24.57 mg GAE/g, and that the amount could rise to 34.89 mg GAE/g with acid hydrolysis, therefore, OF was rich in phenolic compounds. On the other hand, HPH applied before fermentation caused a higher total phenolic content in the samples. This may be a result of the cell walls that are damaged by the homogenization process and causing the release of more phenolic compounds (Quan et al., 2020). In parallel with the change in total phenolic contents, antioxidant activity also increased significantly due to the incorporation of OF and application of HPH. Antioxidant activity was determined as 5.33% in the control sample. Due to their bioactive peptides that possess these activities, probiotic dairy products may show antioxidant activities, as reported by Abdel‐Hamid et al. (2020). The addition of 0.5% and 1% OF increased the antioxidant activity up to 5.66% and 6.83%, respectively. Antioxidant activity is associated with biologically accessible phenolics. Therefore, it can be said that the antioxidant activities of kefir with OF are due to the phenolic compounds contained in the OF. The antioxidant activity results were in line with data previously obtained in similar types of samples (Çakmakçı et al., 2015; Öztürk et al., 2018). The antioxidant activity of kefir samples increased up to 17.22% with the application of HPH. HPH application breaks down the cell walls or cellular structure, allowing more compounds (such as phenolics and vitamin C) to cause antioxidant activities to be released, increasing the antioxidant capacity (Quan et al., 2020).

### 3.5 Water‐holding capacity and textural properties

**FIGURE 3:**
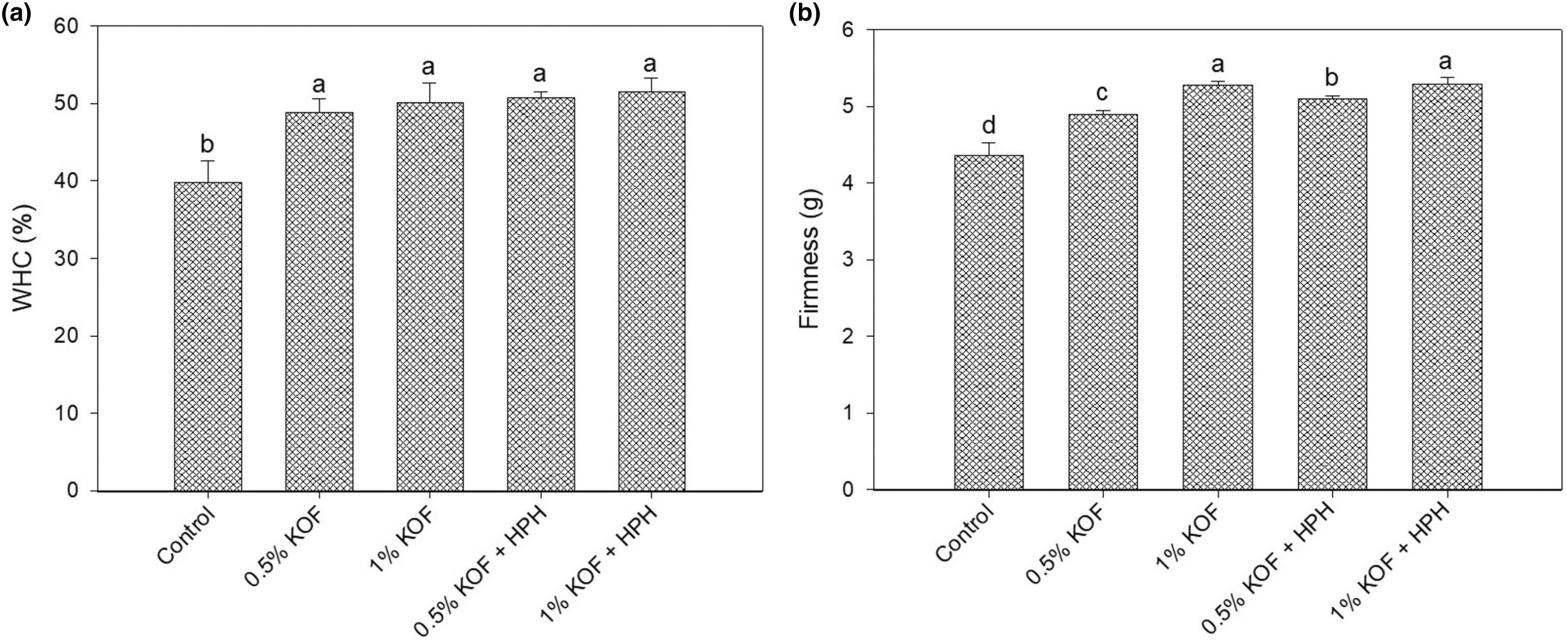
Water‐holding capacity (WHC) and firmness values of kefir samples (0.5% KOF, kefir with 0.5% OF; 1% KOF, kefir with 1% OF; 0.5% KOF + HPH, kefir with 0.5% OF and application of HPH; 1% KOF + HPH, kefir with 1% OF and application of HPH) (different lowercase indicate significant differences between samples (*p* < .05)).

The WHC and textural properties of kefir samples are given in Figure 3A. The lowest WHC (39.74%) was found in the control sample. Compared to the control sample, the WHC of the other kefir samples was found to be significantly higher (*p* < .05) and the highest value was detected as 51.5% for kefir with 1% OF and HPH treatment. However, OF concentration did not make a significant effect on WHC (*p* > .05). The high WHC of kefir samples with OF is due to the increased dry matter and probably high dietary fiber content of OF, which resulted in high WHC of OF, ranging between 372.74% and 430.33% (Sahan et al., 2015). Öztürk et al. (2018) reported that a partial increase was found in the WHC of yogurt samples enriched with 1% and 2% OF. In addition, in the same study, it was noted that syneresis in yogurt samples decreased significantly due to the addition of OF. In another study, it was reported that water retention increased with the use of hazelnut beverage in kefir production, probably due to increased proteins (Atalar, 2019). The amino acid compositions, structures, and surface polarity/hydrophobicity ratios of proteins are the main influences that affect the WHC of foods. There was a partial increase in water‐holding capacity values in kefir samples produced from homogenized milk, but the increase was not statistically significant (*p* > .05). HPH decreased the size of protein, polysaccharides, and fat globules and enhanced fat globules and protein incorporation, causing more water‐holing capacity or fewer phases that separate from fermented milk (Roobab et al., 2023). Levy et al. (2021) reported that no significant change was detected in the WHC of yoghurt‐like products depending on the homogenization pressure. On the other hand, it was stated that nonfat yogurts produced by applying HPH have lower WHC due to their weak gel structure compared to yogurts produced by traditional homogenization, which is probably due to the pressure‐induced fragmentation of casein micelles (Ciron et al., 2010). In contrast, Serra et al. (2007) reported that the WHC of yogurts improved with the applied high pressure >200 MPa, probably resulting in whey protein denaturation as well as protein–protein and protein–fat interactions, which led to improvement of interaction between particles and the formation of a stable gel network that strongly retains water.

The firmness values of the kefir samples are presented in Figure 3B. The firmness value of the control sample was determined as 4.37 g and it is clearly seen that the firmness increased up to 5.27 g with addition of OF. The increment in firmness can be explained by the increase in total dry matter with the addition of OF, which was compatible with the results obtained by Mohan et al. (2020), who stated that firmness was positively correlated with the total solids. Moreover, increasing firmness may be related to the dietary fiber absorbing more moisture due to its high WHC (Sahan et al., 2015), which is supported by WHC results. This observation is supported by some studies that pointed out an increment in firmness for fermented milk products with the addition of different fibers (Garcia‐Perez et al., 2006; Mohamed et al., 2014). Also, McCann et al. (2011) stated that the milk gels with rehydration carrot cell wall particles enhance the gel strength and force it to rupture the gel. On the contrary, in most studies on fortifying fermented dairy products such as kefir and yogurt with plant and agro‐food waste materials, it has been found that the firmness value is reduced, which could be attributed to the destabilization of the gel network. For example, Öztürk et al. (2018) stated that the firmness value in yogurt samples decreases depending on the addition of OF. Almost similar findings were reported by Uruc et al. (2022), who indicated that kefir samples with apricot seed extract have lower firmness than the control sample. Compared to without HPH treatment, a partial increase was observed in the firmness values of kefir samples with the application of HPH, but not statistically different (*p* > .05). Our results agree with the data of Patrignani et al. (2009), who stated that fermented milk from HPH‐treated milk has more compact coagula than the one obtained with only pasteurized milk. Similarly, Ciron et al. (2010) reported that high gel strength and desirable texture for low‐fat yogurt are achieved with milk‐applied HPH technology. This observation could be explained by more casein–casein or casein–fat interactions caused by HPH, and also by the variation in the balance between insoluble and soluble forms of minerals (Patrignani et al., 2007; Swelam, 2018). Moreover, whey proteins are more stable to the applied HPH and can bind with casein and fat to form casein–casein or casein–fat interactions and contribute to the formation of a compact structure (Roobab et al., 2023).

### 3.6 Rheological properties

#### 3.6.1 Steady state

**FIGURE 4:**
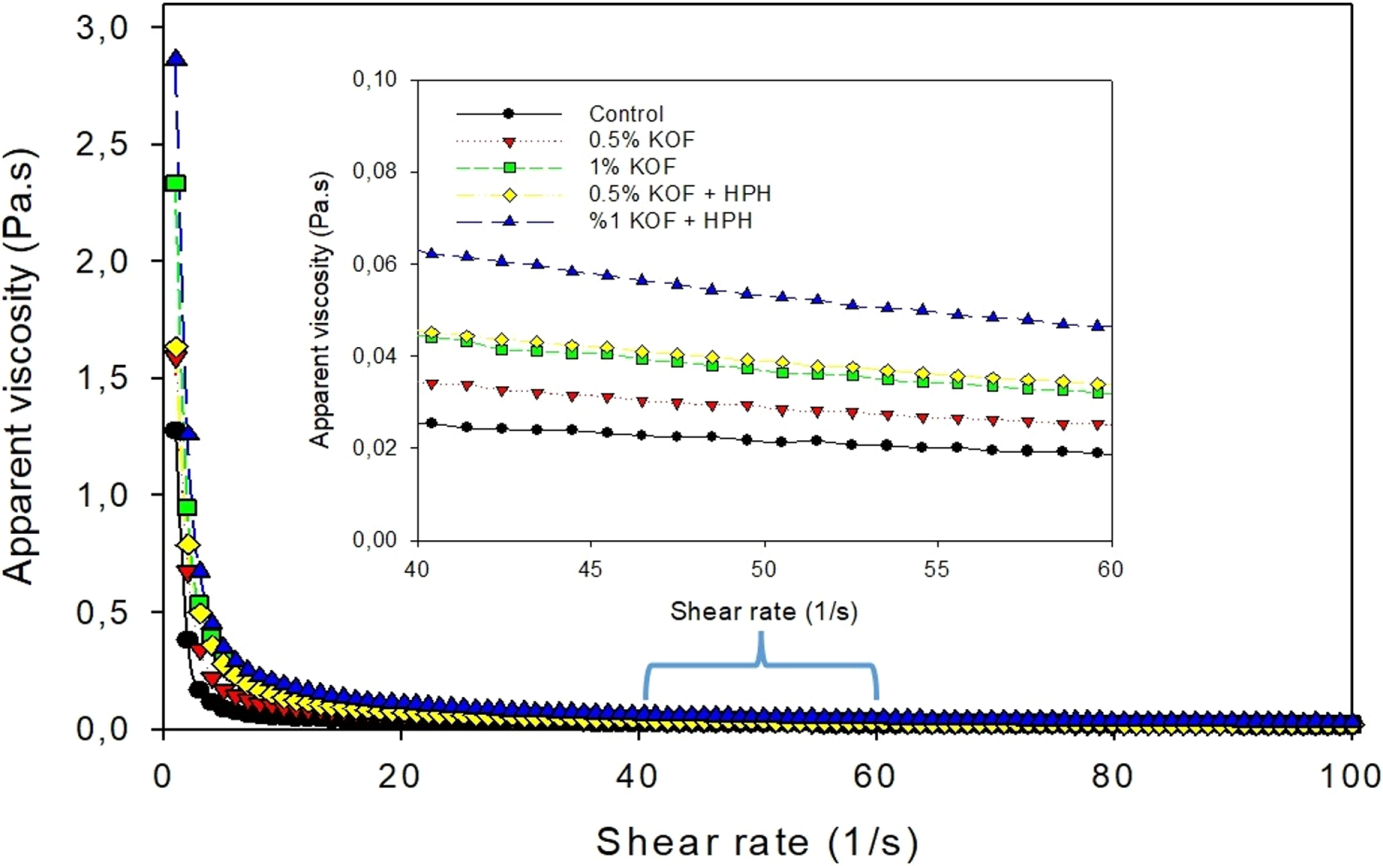
The flow behavior of the kefir samples (0.5% KOF, kefir with 0.5% OF; 1% KOF, kefir with 1% OF; 0.5% KOF + HPH, kefir with 0.5% OF and application of HPH; 1% KOF + HPH, kefir with 1% OF and application of HPH).

In this study, all kefir samples displayed a decrease in apparent viscosity due to the increase in shear rate (Figure 4). They exhibited non‐Newtonian rheological behaviors, a typical behavior for kefirs (Atalar, 2019; Gul et al., 2018) regardless of the addition of OF and HPH treatment, which is common for materials with three‐dimensional structure that is destroyed under the shear forces (Glibowski & Kowalska, 2012; Gul et al., 2018). The Ostwald–de Waele model successfully described the flow behavior of kefir samples due to high determination coefficient (*R* ^2^ > .99). As seen in Table 3, the model parameters with apparent viscosity at 50/s shear rate significantly changed with addition of OF and HPH treatment. The flow behavior index (*n*) of samples was <1, indicating shear‐thinning behavior due to their weak physical bonds and electrostatic and hydrophobic interactions (Ertekin & Guzel‐Seydim, 2010). The highest *n* value (0.306) and lowest *K* value (0.325 Pa.s^*n*^, corresponding to the fluid viscosity) were determined for the control sample, which meant more viscous. The n value significantly decreased with the addition of OF (*p* < .05). Supporting the change in the n value, the *K* value also increased significantly depending on the amount of additional OF, which indicated that the addition of OF led to a thickener structure due to fiber and starch present in the OF. These results are in agreement with the study conducted by Ould Saadi et al. (2020). While HPH treatment did not cause a significant change in the *n* value (*p* < .05), it caused an increase in the *K* value (*p* < .05). Similarly, Tsevdou et al. (2020) reported that the *K* values of beverages increased with high‐pressure treatment.

**TABLE 3:** Viscosity, consistency index, and flow behavior index values of kefir samples obtained from Ostwald–de Waele model system.

Properties	Control	0.5% KOF	1% KOF	0.5% KOF + HPH	1% KOF + HPH
Viscosity (Pa.s)	0.022 ± 0.003^d^	0.03 ± 0.004^c^	0.038 ± 0.002^b^	0.04 ± 0.002^b^	0.048 ± 0.008^a^
*K* (Pa.s^*n*^)	0.325 ± 0.076^d^	0.591 ± 0.044^c^	0.95 ± 0.151^b^	0.866 ± 0.051^b^	1.115 ± 0.091^a^
*n*	0.306 ± 0.059^a^	0.237 ± 0.048^ab^	0.177 ± 0.034^b^	0.213 ± 0.009^b^	0.197 ± 0.019^b^
*R* ^*2*^	0.928 ± 0.018	0.991 ± 0.004	0.977 ± 0.014	0.974 ± 0.011	0.988 ± 0.009

*Note*: Means in the same row with different lowercase letters show significant differences between samples (*p* < .05). *K*, consistency index; *n*, flow behavior index; *R* ^*2*^, determination of coefficient; 0.5% KOF, kefir with 0.5% OF; 1% KOF, kefir with 1% OF; 0.5% KOF + HPH, kefir with 0.5% OF and application of HPH; 1% KOF + HPH, kefir with 1% OF and application of HPH.

As seen in Table 3, the viscosity values of kefir samples were between 0.022 and 0.048 Pa.s at shear rate of 50/s. While control sample had lower viscosity value, the highest value was observed in kefir sample with 1% OF and HPH treatments. The addition of OF and HPH treatments caused a significant increase in the viscosity values of samples (*p* < .05). This was consistent with the results of WHC and firmness shown above, probably relating to high total dry matter and water‐holing capacity of OF. In addition, fibers added in low concentrations can act as attachment points that promote casein aggregation and network formation, resulting in high viscosity (Kieserling et al., 2019). The results were in accordance with another study conducted by Ge et al. (2022), which indicated that fermented milk with 5% of sea buckthorn has the highest viscosity. On the other hand, the formation of insoluble co‐aggregates with high molecular weight due to the denaturation of whey protein during HPH may be the cause of the increase in viscosity values (Massoud et al., 2016). Moreover, casein micelles partially disintegrating during HPH cause a rise in milk particles, which may contribute to the formation of networks and result in a finer network of smaller casein micelles in a three‐dimensional network structure (Serra et al., 2008).

#### 3.6.2 Dynamic shear properties

**FIGURE 5:**
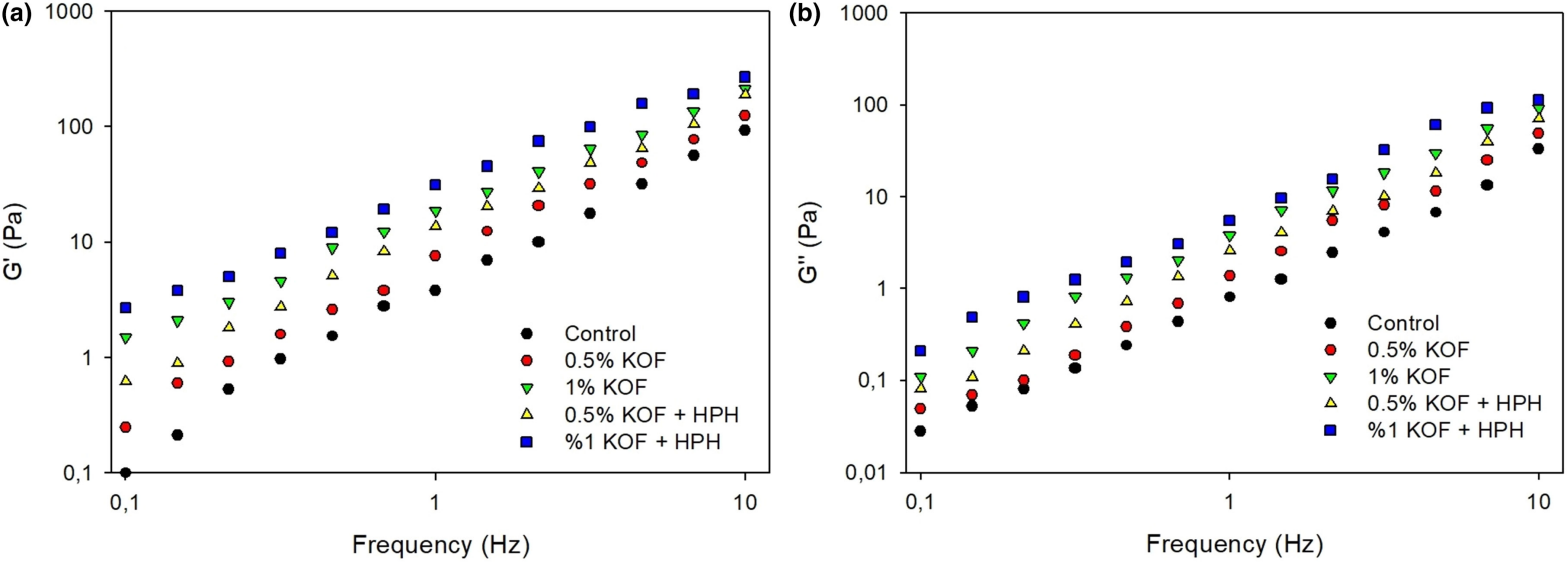
Dynamic shear properties of the kefir samples (0.5% KOF, kefir with 0.5% OF; 1% KOF, kefir with 1% OF; 0.5% KOF + HPH, kefir with 0.5% OF and application of HPH; 1% KOF + HPH, kefir with 1% OF and application of HPH).

The viscoelastic properties of kefir samples determined by dynamic shear tests and the dynamic mechanical spectra of samples are given in Figure 5. Both storage (*G*′) and loss modulus (*G*″), which indicate the degree of solid‐like (elastic) and liquid‐like (viscous) characters, respectively, increased with the increase in angular frequency for all kefir samples, indicating a high‐frequency dependency of gels. For all samples, mechanical spectra showed a typical rheological behavior of kefir samples with *G*′ values higher than the *G*″ values at all oscillatory frequencies (*ω*), which was compatible with other reports (Bensmira et al., 2010; Gul et al., 2018). The *G*′ values of kefirs increased with the addition of OF, indicating that OF was involved in overall gel network formations. This observation was probably due to the presence of higher amounts of total solids in supplemented kefir samples, causing massive crosslinking bridges between casein micelles and protein particles. In addition, polysaccharides found in OF tend to be absorbed onto the protein surface and thus can act as bridges between proteins, resulting in the formation of a strong gel structure that provides higher elastic characteristics (Hamet et al., 2015). Similarly, Raza et al. (2022) reported that the addition of roasted chickpea powder improved the rheological characteristics of yogurts and led to improved structural configurations. In addition, the application of HPH caused an increase in the *G*′ value. It has been suggested by Ayyash et al. (2022) that this observation was based on the interaction between casein and denatured whey proteins that improved the network structure of acid‐induced gel formed during fermentation. Similar findings have been reported by Needs et al. (2000), who stated that high‐pressure treatment enhanced the mechanical characteristics of acid milk gels. So, it can be said that a more stable and stronger gel structure is obtained compared to the obtained control sample with OF supplement and HPH treatment.

*The power‐law model was applied to both moduli of all kefir samples as a power function of oscillatory frequency and the results are given in* Table 4 *. The R* ^*2*^ *values of equations were always higher than 0.987, which meant that power function could be adequately used to describe dynamic shear parameters of kefir samples. K*′ and K″ refer to the solid and viscous characteristics of the kefir samples, respectively, and *n*′ and *n*″ slopes of *G*′ and *G*″ moduli, respectively, which offer information on the frequency dependency of both moduli. In this study, the values of *K*′ and *K*″ increased with addition of OF and HPH treatment compared to control sample, which noted that the elastic and viscous characteristics of acid milk gels were enhanced with the addition of OF and HPH treatments. The *n*′ values (0.882–1.397) were relatively independent of frequency and lower than that of *n*″ values (1.087–2.096) which were more dependent on frequency. Moreover, the slopes of both moduli were significantly affected by addition OF and HPH treatment and the highest values were determined for control sample, indicating that the frequency is more affected.

**TABLE 4:** Dynamic shear parameters of power‐law functions describing G′ and G″ values of kefir samples.

Properties		Control	0.5% KOF	1% KOF	0.5% KOF + HPH	1% KOF + HPH
$G^{\prime }=K^{\prime }{\left(⍵\right)}^{n^{\prime }}$	*K′*	3.749 ± 0.402^e^	7.861 ± 0.341^d^	17.08 ± 0.723^b^	10.10 ± 1.049^c^	36.19 ± 1.509^a^
	*n′*	1.397 ± 0.081^a^	1.198 ± 0.068^b^	1.091 ± 0.106^b^	1.263 ± 0.088^ab^	0.882 ± 0.069^c^
	*R* ^*2*^	0.999 ± 0.003	0.999 ± 0.002	0.999 ± 0.003	0.997 ± 0.007	0.996 ± 0.016
$G^{\prime \prime }=K^{\prime \prime }{\left(⍵\right)}^{n^{\prime \prime }}$	*K*″	0.264 ± 0.027^e^	0.992 ± 0.104^d^	3.779 ± 0.12^b^	1.652 ± 0.208^c^	9.939 ± 1.07^a^
	*n*″	2.096 ± 0.081^a^	1.691 ± 0.073^b^	1.383 ± 0.041^c^	1.638 ± 0.085^b^	1.087 ± 0.097^d^
	*R* ^*2*^	0.997 ± 0.011	0.998 ± 0.009	0.999 ± 0.004	0.998 ± 0.012	0.987 ± 0.031

*Note*: Means in the same row with different lowercase letters show significant differences between samples (*p* < .05). *K′* and *K″*: constants; *n′* and *n″*: frequency exponents; *R* ^2^: determination coefficient; 0.5% KOF, kefir with 0.5% OF; 1% KOF, kefir with 1% OF; 0.5% KOF + HPH, kefir with 0.5% OF and application of HPH; 1% KOF + HPH, kefir with 1% OF and application of HPH.

## 4 CONCLUSION

In this study, the effect of OF on improving the functional, structural, and rheological properties as well as fermentation kinetics of kefir together with HPH treatment was investigated. The addition of OF and application of HPH increased the fermentation rate and therefore shortened the fermentation time by approximately 4 h compared to the control sample. The addition of OF significantly increased the bioactive properties of kefir samples as well as physical properties such as firmness and viscosity. Kefir samples were more elastic than viscous with *G*′ much higher than *G*″ at all frequencies. WHC, which is an important problem for fermented milk products, was significantly enhanced by the addition of OF. HPH also plays an important role in improving the physical properties and WHC of kefir. Additionally, it was determined that the color difference, which was noticeable with the addition of OF, decreased below 3 with the homogenization treatment, which could not be easily detected by the human eye. While the oleaster flour used played an important role in improving the functional properties of kefir samples, its combination with HPH showed a synergistic effect in improving both the structural and functional properties of kefir. Moreover, combining the addition of OF with HPH treatment played an important role in shortening the fermentation time of kefir. In conclusion, the combination of the usage of %1 OF with HPH treatment for functional kefir production may be a promising route. Further studies about the storage stability and consumer preference tests of the final product should be investigated.

## AUTHOR CONTRIBUTIONS

**Latife Betül Gül:** Formal analysis (equal); methodology (equal); writing – review and editing (equal). **Saya Bekbay:** Formal analysis (equal); methodology (equal); writing – review and editing (equal). **Abdullah Akgun:** Formal analysis (equal); methodology (equal); writing – review and editing (equal). **Osman Gul:** Conceptualization (lead); formal analysis (equal); investigation (lead); visualization (lead); writing – original draft (lead); writing – review and editing (equal).

## CONFLICT OF INTEREST STATEMENT

The authors declared no potential conflict of interest with respect to the research, authorship, and/or publication of this article.

## Data Availability

The data that support the findings of this study are available on request from the corresponding author.
